# Impact of Priming and Sprouting on the Quality and Functionality of *Moringa oleifera* Seed Flour

**DOI:** 10.1155/ijfo/3909494

**Published:** 2025-09-26

**Authors:** Ruth-Ann Yaa Frimpong, Nicole Sharon Affrifah, Joris Gerald Niilante Amissah, Joyce Agyei-Amponsah, Josef Kerler, Firibu Kwesi Saalia

**Affiliations:** ^1^ Department of Nutrition and Food Science, University of Ghana, Accra, Ghana, ug.edu.gh; ^2^ Department of Food Process Engineering, University of Ghana, Accra, Ghana, ug.edu.gh; ^3^ Department of Family and Consumer Sciences, University of Ghana, Accra, Ghana, ug.edu.gh; ^4^ The Biotechnology and Nuclear Agriculture Research Institute, Ghana Atomic, Accra, Ghana; ^5^ Nestle Research and Development Centre, Nestle, Konolfingen, Switzerland

**Keywords:** antinutrients, *Moringa oleifera* seed, sensory characteristics, sprouting

## Abstract

This study is aimed at investigating the impact of physical treatments (hydropriming, alkaline‐priming with 0.5% NaOH, and sprouting) as pretreatments to improve the quality of *Moringa oleifera* (*MO*) seed flour for potential use in food products. An in vitro procedure was conducted with steeping medium and sprouting as the experimental factors. Nutrient composition, antinutrients, functional and antioxidative characteristics, sensory attributes, and microbiological quality of the treated seed flour were determined according to standard protocols. Hydropriming (9 h) and sprouting (4 days) increased the protein and fat content by 9% and significantly reduced (*p* < 0.05) the phytic acid, oxalate, and alkaloid content by 42%, 39.9%, and 33%, respectively, compared to untreated *MO* seeds. Conversely, there was a 7.8% increase in tannin content (*p* < 0.05) for hydroprimed and sprouted seeds, while an 18%–25% increase was reported for alkaline‐primed and sprouted seed flour. Sprouting treatments resulted in significant variations (*p* < 0.05) in the functional, total phenolic, and antioxidative properties of the resultant flour. In addition, sprouting increased the anaerobic plate count (APC) by 1–2 log CFU/g while other safety indicators were within the safety limits for sprouted seeds. Principal component analysis (PCA) explained 84.91% of the total variability in the sensory attributes of the samples. Sweet and nutty flavors were developed in the flour when priming was combined with sprouting. Hydropriming *MO* seeds and sprouting can be used as a sustainable method to produce protein‐rich flour with reduced antinutritional factors and bitterness, which can potentially be used as an ingredient for food fortification.

## 1. Introduction

Seed priming is a widely used technique to enhance seed vigour and increase the germination rate by initiating pregermination metabolism, which can reduce seed dormancy under suboptimal conditions [1]. Depending on the aim for seed priming, different seed priming techniques can be adopted, such as hydropriming, chemical priming, osmopriming, and nanopriming, among others ([2]). The simplest method is hydropriming; however, chemical priming using various molarities of sodium hydroxide (NaOH) has been found to be most effective in softening the hard outer coat of seeds [3] to encourage seed water uptake, activate metabolic enzymes to support early seed germination by improving the seeds’ abiotic and biotic stress resistance. The array of physiological changes induced by seed priming has indirect consequences that can be leveraged to improve the nutritive and bioactive potential of seeds, with possible implications for food application. Metabolic events, such as de novo synthesis of proteins and nucleic acids [4], accumulation of enzymes and growth metabolites [5, 6], modulation of reactive oxygen signaling (ROS) pathways [7], synthesis of phenolic compounds through modulation of phenylalanine ammonia‐lyase (PAL) transcripts and activity of PAL enzymes, and activation of antioxidants [8], have been observed in primed seeds, exerting a positive influence on the early growth of seedlings. These increased metabolic events can play an important role in the breakdown of macromolecules and indirectly increase digestible proteins [9] and increase phenolic and flavonoid content with possible antioxidative potential [10, 11], while reducing antinutritional factors [12]. Harnessing the potential of seed priming prior to sprouting is a promising technique geared towards improving the nutritive quality of seeds and is a green method to sustainably produce seed sprouts for plant‐based food applications.

Over the past decade, consumer interest in the use of sprouts in food applications has increased. This is predominantly attributed to the increase in the vegan diet and consumer awareness of the various health‐promoting potential of sprouts. Sprouting is generally considered a green food engineering approach to increase nutrient content, improve flavor, and synthesize secondary metabolites, mainly due to the multitude of physical and metabolic changes catalyzed by enzymes during the sprouting process [13]. Several nutrients, such as proteins, vitamins, and minerals, are reported to increase during sprouting [14]. Additionally, sprouts are a potential natural source of diverse bioactive compounds with various health‐promoting effects for the prevention and treatment of various diseases. These findings support the hypothesis that, beyond meeting nutritional needs, consumption of sprouted seeds may modulate various physiological functions and may play a beneficial role in promoting a state of well‐being, improving health, and reducing the risk of disease. Hence, increasing consumer preference for functional foods may reduce the onset of diet‐related diseases and increase life expectancy. The convergence of these critical concepts has created a dynamic food and beverage market, and sprouted seeds are produced as functional ingredients for incorporation into food products such as yogurt [15], bread [15], pasta [16], beverages [17], and plant‐based meat analogs [18]. Most of these investigations are directed toward exploring the potential of sprouts from underutilized seeds, to harness their nutritional and functional properties while contributing positively to the climate agenda. One such underutilized seeds are *Moringa oleifera* (*MO*) seeds.

*MO* seeds are recommended as a potential food ingredient that must be explored due to their documented health benefits [19, 20]. Moringa foliage is a good source of carbohydrates; essential amino acids such as methionine and phenylalanine; minerals such as calcium, iron, and potassium; and vitamins such as beta carotenoids; vitamins A, B, and C; and alpha‐linoleic acid [21, 22], all of which have a positive impact on reducing cardiovascular, immune, and physiological disorders. Given its many nutritional benefits, moringa has the potential to serve as a functional bioactive‐rich ingredient in fortified food products [23]. However, like most plant‐based foods, the presence of secondary metabolites such as tannins, phytate, alkaloids, and oxalate in *MO* seeds can introduce negative attributes such as bitterness and astringency and impair the digestion and absorption of essential nutrients, which eventually negatively impact sensory quality and consumer acceptability. Hence, to ensure consumer acceptance, the sensory characteristics of *MO* seed flour as influenced by targeted pretreatment, such as sprouting, to reduce secondary metabolites, must be investigated.

The key purpose of this study was to ascertain the effect of seed priming treatments (hydropriming and alkaline‐priming) and sprouting as pretreatments to lower secondary metabolites and elucidate how seed treatment affects the physicochemical and functional characteristics, as well as the microbial quality and sensory descriptors of the resultant flour from *MO* seeds.

## 2. Materials and Methods

### 2.1. Materials

Freshly harvested *MO* seeds (PKM‐2 (Periyankulam‐2)) were purchased from smallholder farmers in Ghana. NaOH, sodium hypochlorite (NaOCl), acetic acid, ethanol, iron chloride, sulphuric acid, hydrochloric acid, sodium carbonate (Na_2_CO_3_), 2,2‐diphenyl‐1‐picrylhydrazyl (DPPH), methanol, calcium chloride, dichloran rose bengal chloramphenicol (DRBC) agar, tryptic soy agar, violet red bile agar, baird parker agar, plate count agar, methanol, Folin‐Ciocalteu phenol regent, ethyl acetate, boric acid, acetone, and petroleum ether were purchased from Sigma Aldrich, Merck, and Oxoid Ltd. Basingstoke, Hampshire, United Kingdom.

### 2.2. Experimental Design

The effect of the treatment parameters on MO seed flour was carried out following a 2 × 2 factorial design. The factors were seed priming agent (i.e., water or 0.5% NaOH solution) and sprouting (4 days of sprouting and not sprouting). A factorial matrix with the coded factors is presented in Table 1.

**Table 1:** Factorial design for physical treatment of *MO* seeds.

**Treatment code**	**Treatment**	**Soaking medium**	**Sprouting**
NSNS	Not soaked, not sprouted	0	−1
SWS	Soaked in water and sprouted	+1	+1
SNS	Soaked in 0.5% NaOH and sprouted	−1	+1
SN	Soaked in 0.5% NaOH only	−1	−1
SW	Soaked in water only	+1	−1

### 2.3. Hydration Capacity (HC) of *MO* Seeds

This was determined according to the AACC method 56‐35.01 [24]. The HC (g/seed) of *MO* seeds was estimated on 100 uniformly sized and damage‐free seeds as the gain in seed weight after soaking for a predetermined period (3, 6, 9, and 12 h) in 50‐mL distilled water. The HC (%) was calculated using the formulas by da Silva et al. [25] as shown in Equation (1) and expressed as the percentage increase in seed weight. All treatments were performed in triplicate, and results were expressed as mean values with associated standard deviations.

(1) HC%=W1−W0W0×100,

where *W*_1_ is the weight of seeds after soaking in water and *W*_0_ is the weight of seeds before soaking in water.

### 2.4. Germination Percentage (GP) of *MO* Seeds

This was determined according to Farahzety et al. [26], with modifications. GP was estimated as the number of seeds that germinated within an allocated number of seeds (Equation 2) and expressed as a percentage.

(2) Germination rate %=G1Ts×100,

where *G*_1_ is the number of germinated seeds and *T*_s_ is the total number of seeds.

### 2.5. Radicle Length of *MO* Seeds

Radicle length was determined following the method by Zhang et al. [27] with modifications. From each seed treatment, four replicates of 30 seeds were selected and placed in a sterilized petri dish (90‐mm diameter and 15‐mm height) lined with filter paper (Whatman No. 1) and moistened with 2.5 mL of distilled water. The petri dish was placed in the germinator (Seedburo‐164918401, United States) for 4 days at 23°C–28°C and 98*%* ± 2*%* humidity. Periodically, the seeds were moistened with distilled water to prevent dehydration. At the end of the germination experiment, a digital vernier caliper was used to determine the radicle length of 20 randomly selected *MO* seed sprouts, and the mean length values were reported in centimeters.

### 2.6. Preparation of *MO* Seed Flour

**Figure 1:**
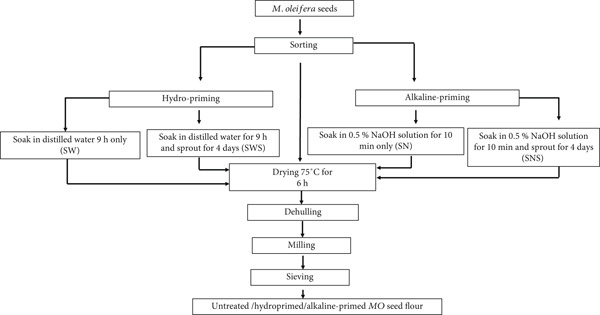
Flow chart to produce untreated, hydroprimed, and alkaline‐primed *M. oleifera* seed flour.

The flow diagram depicting the preparation of *MO* seed flour is seen in Figure 1. The seeds were cleaned manually to remove broken seeds, dust, and other extraneous materials. The grains were sterilized by soaking in 200‐ppm NaOCl for 10 min, rinsed several times under running water, and drained before steeping. The cleaned seeds were divided into four batches of 300 g and were steeped in distilled water (1.5 L) [28] for 9 h or in 0.5% NaOH solution for 10 min. The steeped seeds were spread on sterile wet jute bags to sprout at room temperature (23°C–28°C) and 98*%* ± 2*%* humidity for 4 days in a germinator (Seedburo‐164918401, United States). Sprouting was conducted in a dark room to prevent the interruption of light. Sprouting was established when rupture of the tegument and visible elongation of the coleoptile and coleorhiza were observed. Ungerminated seeds were not included in sampling. At the end of the designated time, the germinated seeds were dried in a vacuum oven (Genlab Oven, Model: DP/100/SS/F/DIG) at 75°C for 6 h to remove moisture and halt germination. Rootlets and shoots of the seed were separated from the kernels by rubbing the germinated grains in a 0.6‐mm sieve. The sprouted and unsprouted seeds were dehulled and milled separately into fine flour with a spice mill to pass through a 0.4‐mm sieve. The *MO* seed flour was stored in sterile conditions.

### 2.7. Physical and Chemical Characteristics of *MO* Seed Flour

#### 2.7.1. Proximate Composition of *MO* Seed Flour

The ash (923.03), lipid (920.85), crude protein (*N* × 6.25) (920.87), crude fiber (978.10), and moisture content (950.46) of the *MO* seed flour were determined according to the Association of Official Analytical Chemists method [29]. The carbohydrate content was determined by difference, and the total energy was calculated using the Atwater factor method.

#### 2.7.2. Monomeric Sugar Analysis by High‐Performance Liquid Chromatography (HPLC)

The treated *MO* seed flour was prepared for monomeric sugar analysis using the method described by Van Wychen and Laurens [30] with modifications. The flour was sequentially hydrolyzed with 72% sulfuric acid at 30°C for 1 h and 4% sulfuric acid at 121°C for 1 h. The supernatant was filtered using 0.2‐*μ*m syringe filters into 1.5‐mL screw vials for monomeric sugars using HPLC.

Monomeric sugar analyses were performed on a Shimadzu LC10/20 HPLC equipped with a Refractive Index detector and an Aminex 87H column (Bio‐Rad Laboratories, United States). HPLC was operated at a column temperature of 60°C and a detector temperature of 55°C with 0.005 M sulfuric acid as the mobile phase at a flow rate of 0.6 mL/min. The sample injection volume was 10 *μ*L per sample. HPLC was precalibrated using high‐purity standards of various monomeric sugars.

#### 2.7.3. Determination of Mineral Content

The method outlined by Bhat et al. [31], with modifications, was used for mineral determination. Then, 1 g of each sample was weighed, ashed at 550°C for 2 h, and then allowed to cool. The ash was transferred into a 250‐mL beaker, and 15 mL of H_2_SO_4_ and 5 mL of concentrated nitric acid were added. The solution was boiled, and 10 mL of distilled water was added to the beaker. The solution was filtered into a 100‐mL volumetric flask and diluted to the mark. The mineral content of the digested sample was analyzed at 589.0–589.6 nm using an atomic absorption spectrophotometer (PG instrument AA500). The concentrations of macrominerals, that is, phosphorus (P), and calcium (Ca) and microminerals, i.e., iron (Fe) and zinc (Zn), in each sample were determined. The calibration curves of absorbance values against appropriate concentrations of each mineral were estimated as milligrams per 100 g of sample.

#### 2.7.4. Bulk Density of Flour

The flour sample (50 g) was placed in a 100‐mL measuring cylinder. The cylinder was tapped continuously until a constant volume was obtained. The bulk density (g/cm^3^) was calculated as the weight of flour (gram) divided by flour volume (cubic meter).

#### 2.7.5. pH Determination

The pH of the treated *MO* seed flour was determined according to the method outlined in AOAC 991‐12. Approximately 1 g of each sample was introduced into centrifuge tubes and mixed with 10 mL of distilled water. The mixture was stirred for 30 min using a vortex mixer and centrifuged at 4500 g for 15 min. The pH of the supernatant was determined using a calibrated pH meter at room temperature (25°C).

#### 2.7.6. Determination of Phytic Acid

Phytate content was determined according to Karanja et al. [32] with modification. Then, 10 mL of 3% H_2_SO_4_ was added to 0.5 g of *MO* seed flour. The mixture was then filtered into 50‐mL centrifuge tubes and placed in a boiling water bath for 5 min, followed by the addition of 3 mL of iron (III) chloride solution (6 mg ferric iron per mL in 3% H_2_SO_4_) to precipitate ferric phytate. To convert ferric phytate to sodium phytate, 3 mL of 1.5 N NaOH was added, and the volume was increased to 30 mL using distilled water. The mixture was heated in a boiling water bath for 30 min to precipitate ferric hydroxide. The mixture was then cooled to an ambient temperature, centrifuged, and the supernatant was transferred into a 50‐mL volumetric flask. This was followed by rinsing the precipitate with 10 mL of distilled water, centrifuged again, and the supernatant was added to the content of the volumetric flask. Then, 2 mL of ferric ammonia sulfate then was added to the filtrate as an indicator, and the mixture was titrated with 0.01 M of standard ammonia thiocyanate solution until a brick red/reddish brown endpoint was reached, indicative of the formation of the ferric thiocyanate complex. The results were reported as milligrams per gram of flour and based on the formula below, modified from Latta and Eskin [33]:

(3) Phytic acid mg/g=F netotal3+−volume NH4SCN×NH4SCN4×660×1000Msampleg,

where ^n^Fe ^3+^_(total)_ is the total moles of ferric ions added, [NH_4_SCN] is the concentration of NH_4_SCN (mol), Volume of NH_4_SCN is the titer value (mol/L), molar mass of phytic acid = 660, and *M*_sample_ is the mass of the sample.

#### 2.7.7. Determination of Tannin Content

The tannin content was determined according to the method described by Pandey et al. [34] with modification. Firstly, 1 g of the sample was dispersed in 50‐mL methanol and stirred for 20–28 h. The mixture was centrifuged (3000 rpm and 10 mins) to obtain the supernatant. Then, 1 mL of the supernatant was added to 5 mL of vanillin hydrochloric acid reagent (equal volumes of 8% hydrochloric acid in methanol and 4% vanillin in methanol). A standard tannic acid solution (0–100 mg/100g) was prepared and used as the reference standard. The absorbance of the mixtures was measured after 20 m at 492 nm using a UV‐Vis spectrophotometer (Cole‐Parmer, S/N: KPX15031503025), and tannin content was expressed as milligrams of tannin per 100 g of sample using the formulas in Equation (4). All results were an average of triplicate readings.

(4) Tannin content=Absk×50Msample/100,

where Abs is the absorbance of the sample, *k* is the constant, and *M*_sample_ is the mass of the sample (g).

#### 2.7.8. Determination of Oxalate Content

Oxalate was determined according to Adeleke et al. [35]. Briefly, 2 g of the sample was dispersed in 190‐mL distilled water, acid‐digested in 10 mL (6 M HCl for 2 h), and centrifuged (2000 rpm, 10 min). Subsequently, 50 mL of the supernatant was filtered, and the solution was titrated against concentrated ammonia solution until the methyl red indicator transitioned to a faint yellow color. The solution was heated to 90°C, and 10 mL of 5% (*w*/*v*) CaCl_2_ solution was added to precipitate oxalate overnight. The precipitate was then washed to remove the excess calcium and washed into a 100‐mL conical flask with 10‐mL H_2_SO_4_ (25% *v*/*w*) and 15‐mL distilled water. The solution was heated to 90°C and then titrated against 0.1 M KMnO_4_ to a pink‐colored endpoint. Oxalate levels were calculated by multiplying the KMnO_4_ titer value by 0.1125. All determinations were carried out in triplicate and expressed as milligrams of oxalate per 100 g of sample.

#### 2.7.9. Determination of Alkaloid Content

Alkaloid content was determined according to the method of Waseem et al. [36]. Then, 5 g of the sample was dispersed in 50 mL of a 10% acetic acid solution in ethanol and filtered using Whatman filter paper No. 41. The filtrate was titrated against conc. ammonium hydroxide, followed by a 1% solution of ammonium hydroxide. The alkaloid precipitates were oven‐dried at 55°C for 30 min, and the alkaloid content was calculated gravimetrically and expressed as milligrams of alkaloid per 100 g of sample.

### 2.8. Functional Characteristics of Treated and Untreated *MO* Seed Flour

#### 2.8.1. Water Holding Capacity (WHC)/Oil Holding Capacity (OHC)

The water and oil absorption capacities of the flour samples were determined using the methods described by Beuchat [37]. Then, 5 g of treated or untreated *MO* seed flour was weighed and hydrated with 15 mL of distilled water/refined sunflower oil at 25°C for 1 h with manual stirring at 10‐min intervals. The supernatant was drained with Whatman No. 2 filter paper with slight suction, and the retentate was reweighed. The supernatant was decanted, and the sample was reweighed. The amount of water retained in the sample was recorded as the weight gained and then taken as the water absorbed. An identical method using sunflower oil as a dispersant was adopted to measure OHC, and OHC was expressed as grams of oil held per gram of seed flour.

The absorption capacity was calculated as follows:

(5) WHC=W1/Ws,OHC=W2/Ws,

where is the weight gain upon hydration, *W*_1_ is the weight of water absorbed, *W*_2_ is the weight of oil absorbed, and *W*_s_ is the weight of dry sample.

#### 2.8.2. Extraction and Determination of Phenolic Compounds

The extraction of phenolic compounds was according to the method described by Sardabi et al. [38] with modifications. The phenolic content of defatted *MO* seed flour was extracted with ethanol:water (50:50 *v*/*v*) at a ratio of 1:10 (*w*/*v*) at 25°C and 200 rpm for 24 h. Subsequently, the slurry was centrifuged at 2000 g for 20 min. The extracts were then filtered through Whatman filter Paper No. 1. Supernatants were recovered, and TPC was measured using the Folin‐Ciocalteu method. The extract (20 mL) was mixed with 1.60 mL of distilled water and 100 mL of 0.2 M Folin‐Ciocâlteu phenol reagent. After 8 min of incubation at room temperature, 300 mL of 20% Na_2_CO_3_ solution was added, and the mixture was stored at 40°C in a shaking water bath. After 30 min, the absorbance of the sample solutions was measured at 760 nm using a UV‐Vis spectrophotometer (Cole Parmer, model: S/N: KPX15031503025, United States). The polyphenol content of the extract was expressed as milligrams of gallic acid equivalents (GAE) per gram of dry sample, calculated from the prepared standard curve with 0.1–0.8 mg/gallic acid (GA).

#### 2.8.3. Scavenging Activity on DPPH Free Radicals

The oxygen scavenging ability of all treated *MO* seeds was examined according to Safdar et al. [39] with modifications. The sample (0.05 g) was dispersed in deionized water at a ratio of 1:10 (*w*/*v*) and mixed with 2.0 mL of 0.1 mM DPPH in methanol, stirred, and stored in the dark at room temperature for 30 min. Solutions were also prepared without adding *MO* seed flour samples (control) or DPPH (blank). Absorbance was measured at 517 nm using a UV‐Visible spectrophotometer. All assays were performed in triplicate. The DPPH radical scavenging activity was calculated using the following formula:

(6) Scavenging activity rate=Sa−BaCa×100,

where *S*_*a*_ is the absorbance of the sample at 517 nm, *B*_*a*_ is the absorbance of the blank, and *C*_*a*_ is the absorbance of the control.

### 2.9. Determination of Microbiological Quality and Safety of Treated *MO* Seeds

#### 2.9.1. Enumeration of Yeasts and Molds

Yeasts and molds were enumerated in accordance with ISO 21527‐1: 2008 [40] using the spread plate method on DRBC agar (Oxoid CM0727), pH 5.6, containing chloramphenicol supplement to prevent bacterial growth. The plates were then incubated at 25°C for 3–5 days. Quantitative data were log‐transformed and reported as log CFU per gram of flour.

#### 2.9.2. Enumeration of *Escherichia coli* (*E. coli*)

*E. coli* was determined by the pour plate method on tryptic soy agar (Oxoid CM0131), at pH 7.3, according to the Nordic Committee of Food Analysis [41]. Serial sample dilutions were prepared and inoculated on sterile petri dishes. Molten TSA was poured onto the petri dish and swirled for an even distribution between the inoculum and agar. The plates were incubated at room temperature and allowed to set for 1–2 h. Afterwards, Violet Red Bile Agar at pH 7.4 was overlaid on the plate and allowed to set at room temperature. The plates were then incubated at 44°C for 24 h for *E. coli* enumeration. Results were recorded as log colony‐forming units (log CFU/g) of flour, following the log transformation of quantitative data.

#### 2.9.3. Enumeration of *Staphylococcus aureus* (*S. aureus*)

*S. aureus* was determined using the spread plate method on Baird Parker Agar (Oxoid CM0275), containing egg yolk tellurite emulsion (SR54). Suspected colonies were confirmed to be coagulase‐positive in a rabbit coagulase plasma (C14389) according to the method described by the Nordic Committee of Food Analysis [42]. Each serial dilution (0.1 mL) was inoculated onto the surface of prepared Baird Parker agar in a petri dish. Using a sterile spreader, the inoculum was spread uniformly on the surface of the agar. The inoculum was allowed to dry at room temperature and was incubated at 37°C for 48 h. Results were recorded as log colony‐forming units (log CFU/g) of flour, following log transformation of quantitative data.

#### 2.9.4. Enumeration of Aerobic Mesophiles

Aerobic mesophilic bacteria were quantified following the pour plate technique as described by the Nordic Committee on Food Analysis [43]. Serial decimal dilutions (10^−1^–10^−6^) of flour samples were prepared under aseptic conditions. A 1‐mL aliquot from each dilution was transferred into sterile Petri dishes, to which approximately 15–20 mL of molten plate count agar (Oxoid CM0325), tempered to 45°C, was added. After gentle mixing, plates were allowed to solidify at ambient temperature and subsequently incubated at 30°C for 72 h. Plates exhibiting 25–250 colonies were selected for enumeration to ensure statistical reliability. Microbial counts were expressed as log colony‐forming units per gram (log CFU/g) following logarithmic transformation of raw data.

### 2.10. Sensory Characteristics of *MO* Seed Flour

Quantitative descriptive analysis (QDA) was employed to determine differences in the sensory characteristics of the treated and untreated *MO* seed flour in its raw state. Sensory assessments of the samples were performed using a trained panel consisting of 11 members. All panelists passed the basic taste test, odor test, and color vision tests. For attribute generation, the panelists were given both treated and untreated *MO* seed flour, and references for each attribute were provided (Tables 2 and 3). Additionally, the assessors were trained to evaluate the sensory descriptors identified at various degrees of intensity. The panelists evaluated the attributes of *MO* seed flour using a structured 10‐point scale, where 1 represented *no perception of the attribute* and 10 represented *a very intense perception of the attribute*. Samples (i.e., 5 g of *MO* seed flour) were presented to each assessor in three‐digit random number–coded plastic containers, covered with lids. Along with the samples, the panelist received a cup of room temperature mineral water and dry crackers for cleansing their palate. Assessments were carried out at the BNARI sensory laboratory with individual booths with appropriate ventilation, white lighting, and controlled temperature. The rights and privacy of all participants were considered during the execution of the research and explicitly outlined in a document signed by all participants before the commencement of the study.

**Table 2:** Evaluated attributes and their sensory descriptors.

**Aroma**	**Flavor**	**Texture**	**Aftertaste**
Herby	Rancid	Oily	Astringent
Nutty	Astringent	Smooth	Oily
Rancid	Nutty	Gritty	Sweet
Fermented	Beany	Melty	Bitter
	Chili		Chili
	Sour		
	Sweet		
	Bitter		

**Table 3:** Definition of sensory descriptors.

**Attribute**	**Definition**	**Reference**
*Texture*		
Smooth	Softness, gentle sensation in the mouth, sensation of substance‐free from lumps	Milk pudding
Oily	The feeling of the mouth coating associated with eating vegetable oil or butter	A stick of butter
Gritty	The perception of small, hard particles reminiscent of sand	Corn meal
Melty	A feeling associated with the dissolving of whipped cream on the tongue	Whipped cream
*Flavor*		
Bitter	Bitterness associated with caffeine	0.5% caffeine solution
Sour	Taste associated with unflavored, unsweetened yogurt	Plain yogurt
Nutty	Slightly sweet, brown, woody, oily, musty nuts and beans and grains	Untoasted almond
Sweet	Taste on the tongue associated with sugars	5% sucrose solution
Chili	Tingling feeling on the tongue	Spice or pepper
Beany	Taste associated with uncooked green peas or beans	Fresh green peas
Astringent	A feeling of a puckering or a tingling sensation on the surface and/or edge of the tongue and mouth	0.03% alum solution
Rancid	Aroma associated with oxidised oil, melting crayon wood vanish or play dough	Wet cardboard, aged vegetable oil
*Aroma*		
Herby	Fresh, slightly sour aroma associated with newly cut grass	Green tea leaves
Rancid	Aroma associated with oxidized oil	Aged vegetable oil
Fermented	Aroma associated with fermented raw corn dough	Raw corn dough
Nutty	Slightly sweet, brown, woody, oily, musty nuts and beans and grains	Untoasted almond
*Aftertaste*		
Bitter	Bitterness associated with caffeine	0.5% caffeine solution
Sweet	Taste on the tongue associated with sugars	5% sucrose solution
Sour	Taste associated with unflavored, unsweetened yogurt	Plain yogurt
Chili	Tingling feeling on the tongue	Spice or pepper
Astringent	A feeling of puckering or a tingling sensation on the surface and/or edge of the tongue and mouth	0.03% alum solution

### 2.11. Statistics

Data obtained from the analysis of physicochemical, functional, phenolic, and antioxidative characteristics were subjected to an analysis of variance (ANOVA). All tests were performed in triplicate, and the data were subjected to one‐way ANOVA using the SPSS 19 software (SPSS Inc., Chicago, IL, United States). Significant mean values were determined at a 95% confidence level (*p* ≤ 0.05) using Tukey’s HSD test. Assumptions of normality were evaluated via visual inspection of histograms and Levene’s test for variance homogeneity. Principal component analysis (PCA) was performed on standardized *z*‐scores of sensory data to assess sample clustering and variable contribution among all sensory data using XLSTAT. Graphs and tables were generated using Microsoft Excel (Version 16.0).

## 3. Results and Discussion

### 3.1. Effect of Imbibition Time on the HC and Moisture Content of *MO* Seeds

Seed imbibition dynamics can be a useful index for selecting seed hydration pretreatment for efficient germination; therefore, seed water status is essential for sprout production. Accordingly, this study was conducted to determine the water content of *MO* seeds as a time‐dependent trait to explore its water saturation points. The results are presented in Table 4.

**Table 4:** The impact of imbibition time on hydration capacity and moisture content of *MO* seeds.

**Imbibition time (hours)**	**Hydration capacity (g/seed)**	**Moisture content (g/100g)**
0	Nil	1.49 ± 0.21^e^
3	0.60 ± 0.01^c^	2.94 ± 0.21^d^
6	1.07 ± 0.00^b^	4.99 ± 0.27^c^
9	1.46 ± 0.01^a^	12.83 ± 0.42^b^
12	1.45 ± 0.01^a^	13.95 ± 0.22^a^

*Note:* Data represent the mean of three independent experiments (*n* = 3). Means with different superscripts in a column indicate significant differences (*p* < 0.05). Data were generated from the hydroprimed seeds.

**Figure 2:**
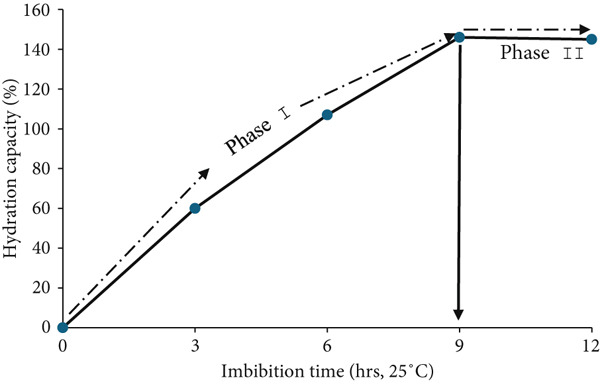
Imbibition pattern of *M*. *oleifera* seeds at different time points.

The HC and moisture content of *MO* seeds varied with the imbibition time. Overall, imbibition time was positively correlated to the HC and moisture content of *MO* seeds (*p* < 0.05, *r* = 0.99). The water uptake mechanism of seeds begins with imbibition through the micropyle to the cell wall, then to reserve polymers, and finally to the seeds’ inner structure [8]. Macrostructures such as proteins, starch, and fibers are hygroscopic; thus, they gradually absorb water over time and retain it due to their high WHC [44]. This mechanism reaches an equilibrium when the maximum seed volume is achieved [45, 46]. For this study, the typical results in the imbibition pattern as a function of time show a characteristic asymptotic curve, as observed in Figure 2.

The phase transitions between Phase I and Phase II were distinguishable as explained by Nonogaki et al. [47]. Between 0 and 9 h of imbibition, there was a rapid increase in the water uptake; however, above 9 h of imbibition, a plateau was reached. This is an indication of water saturation. This phase is characterized by cell reserve mobilization in preparation for cell expansion and germination. Similar imbibition patterns were also reported for cowpea [48]. At maximum hydration, there is an increase in mitochondrial activity, which is coupled with biogenesis and de novo protein synthesis, which culminates in germination [47, 49]. Based on the data from this study (Table 4), it was observed that imbibition for 9 h increased (i.e., > 100%) in moisture content, which represents complete hydration of the *MO* seed necessary to initiate cell metabolic processes that support cell expansion to promote germination.

### 3.2. Effect of Hydropriming and Alkaline‐Priming on Germination Rate and Radicle Length of *MO* Seeds

**Figure 3:**
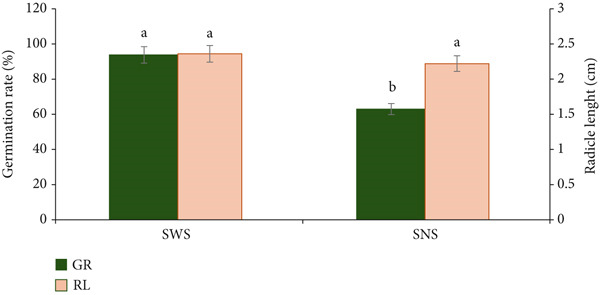
Germination rate (GR) and length of radicle (RL) of MO seeds following hydropriming and sprouting (SWS) and alkaline priming and sprouting (SNS). Bars with different superscripts indicate significant differences (*p* < 0.05).

The effect of the different seed priming agents on germination rate and radicle length was determined and represented in Figure 3. Based on the results in Figure 3, the germination rate of *MO* seeds was significantly affected by the seed priming medium. SWS had the highest germination rate of 92.9 ± 0.38*%*, which was significantly different (*p* < 0.05) from SNS, which was 62.96 ± 0.37*%*. This indicates that hydropriming successfully enhanced the germination rate by 29.94% when compared with alkaline‐primed seeds. This observation is aligned with studies by Farahzety et al. [26] and Nouman et al. [50], who reported a 63% germination rate for hydroprimed *MO* seeds. A similar study by Coello et al. [51] recommended soaking *MO* seeds for 18 h prior to sprouting; however, under the current study conditions, soaking for 9–12 h was sufficient to reach the maximum seed HC (Table 4) to facilitate *MO* seed sprouting. This inevitably may reduce the total processing time and eventually the total cost of producing *MO* seed sprouts. Some studies also suggested that, as compared to other seed priming strategies, hydropriming improved seed germination through the expression of transmembrane proteins called aquaporins [52–54]. Specifically, aquaporins trigger the modulation of plant intercellular water transport to reduce cell wall turgor and activate cell wall hydrolase activity to loosen the cell wall for radicle emergence, among others, resulting in higher values of germination rate [8, 55, 56]. A culmination of all these biochemical events supports the early germination of hydroprimed seeds. Generally, hydropriming of *MO* seeds for 9 h or priming with 0.5% NaOH for 10 min supports seed germination and breaking seed dormancy, unlike untreated seeds, which cannot germinate without pretreatment. Furthermore, the soaking medium did not significantly (*p* > 0.05) affect the length of the emerging radicle, which ranged between 2.22 ± 0.07 and 2.36 ± 0.20 cm (Figure 3) for alkaline‐primed and hydroprimed seeds, respectively. This shows that the seed emergence and establishment were unaffected, signifying that the use of hydropriming or alkaline‐priming pretreatments outlined in this study can result in effective *MO* seed germination.

### 3.3. Effect of Seed Priming and Sprouting on the Physicochemical Characteristics of *MO* Seed Flour

The macronutrient content of primed and sprouted *MO* seeds was determined through proximate analysis to establish the effect of seed priming and sprouting on the nutritional quality of *MO* seed flour. This study reveals that crude protein and crude fat are the most abundant macronutrients in *MO* seed flour and represent 41.44% and 33.41%, respectively (Table 5).

**Table 5:** The proximate characteristics of treated *MO* seed flour.

**Parameters**	**Treatments**				
	**NSNS**	**SW**	**SN**	**SWS**	**SNS**
***g/100g DM; p<0.05***					
Crude Protein	41.44± 1.01^c^	41.03±0.45^c^	40.66±0.16^c^	45.27±0.01^a^	43.91±0.73^b^
Crude Fat	33.41±0.61^c^	32.55±0.01^c^	33.06±0.51^c^	36.30±0.70^a^	34.79±0.39^b^
Crude Fibre	7.63±0.63^a^	5.30±0.24^b^	5.40±0.08^b^	4.29±0.26^c^	4.29±0.18^c^
Total Ash	3.40±0.03^a^	3.32±0.00^b^	3.12±0.01^c^	3.37±0.01^ab^	3.35±0.08^ab^
Carbohydrate	10.80±0.79^b^	15.26±0.47^a^	14.97±0.56^a^	7.19 ± 2.39^c^	8.52±0.96^c^
Glucose	1.57±0.02^bc^	1.47±0.04^c^	1.64±0.05^b^	1.65±0.04^b^	1.97±0.09^a^
Xylose	0.80±0.06^ab^	0.86±0.03^a^	0.82±0.05^a^	0.67±0.00^c^	0.73±0.00^bc^
Mannose	0.97±0.02^bc^	0.97±0.05^bc^	0.92±0.04^c^	1.14±0.00^a^	1.02±0.02^b^
Arabinose	1.45±0.03^a^	1.37±0.02^b^	1.26±0.01^c^	1.01±0.04^d^	1.04±0.03^d^
Energy(kcal/100g)	509.70±5.60^c^	518.13±0.59^b^	519.94±2.69^b^	530.60±4.21^a^	522.82±1.67^b^
***Mineral composition mg/100g DM; p<0.05***					
Iron	12.53±0.39^a^	13.21±1.06^a^	2.27±0.00^c^	6.26±0.47^b^	2.90±0.71^c^
Phosphorus	1184.36±0.01^a^	1148.73±0.06^a^	234.58±0.00^c^	947.88±0.00^b^	934.47±0.00^b^
Calcium	336.67±0.00^a^	310.00±0.03^ab^	290.00±0.00^b^	243.33±0.00^c^	270.00±0.00^bc^
Zinc	1.44±0.19^a^	1.28±0.10^ab^	0.58±0.01^c^	1.07±0.27^b^	1.33±0.09^ab^
***Antinutrient mg/100g DM; p<0.05***					
Tannin	2.64±0.10^c^	2.38±0.09^d^	3.3±0.29^a^	2.85±0.05^b^	3.14±0.08^a^
Alkaloid	4.08±0.03^a^	2.90±0.30^b^	3.99±0.23^a^	2.72±0.02^b^	4.03±0.01^a^
Oxalate	2.78±0.01^a^	1.93±0.00^b^	0.66±0.05^e^	1.67±0.00^c^	1.09±0.02^d^
Phytic acid	70.00±0.00^a^	56.67±0.00^b^	50.00±0.00^b^	40.00±0.00^c^	50.00±0.00^b^

*Note:* Data represent the mean of three independent experiments (*n* = 3). Means with different superscripts in the row indicate significant differences (*p* < 0.05).

Abbreviations: NSNS (Control): Not soaked, not sprouted; SW: soaked in water; SN: soaked in 0.5% NaOH solution; SWS: soaked in water with sprouting; SNS: soaked in 0.5% NaOH solution with sprouting.

#### 3.3.1. Protein Content

Flour processed from sprouted *MO* seeds had a crude protein content within the range of 43.91 ± 0.73*%*–45.27 ± 0.01*%* and crude fat content between 34.79 ± 0.39*%* and 36.30 ± 0.70*%* (Table 5). The values for crude protein for all seed treatments in this study were higher than those reported by Chinma et al. [57]. This variation may be due to the geographical location and growth conditions of the *MO* seeds. When compared with the NSNS, combining priming *MO* seeds with sprouting increased the crude protein by 5–9% (*p* < 0.05) in SNS and SWS, respectively. Coello et al. [51] reported similarly and indicated a 13.7% increase in protein content for *MO* seeds after hydropriming for 18 h and sprouting for 60–96 h at 32°C. Also, Perveen et al. [58] reported an over 100% increase in soluble proteins after barley seeds were primed with NaOH prior to sprouting. The increase in protein content can be attributed to the synthesis of de novo proteins, which also contributes to the overall protein content. Furthermore, seeds that were only primed without sprouting (SW and SN) recorded similar crude protein content (41.03 ± 0.45 and 40.66 ± 0.16, respectively) when compared with the NSNS. From these observations, combining priming with sprouting can be an alternative approach to improve the protein content of *MO* seed flour.

#### 3.3.2. Crude Fat

The crude lipid in *MO* seed was 33.41 ± 0.61*%* (NSNS), and treatments such as SN and SW did not cause a significant difference in its content. However, there was a 4.1%–8.65% increase in crude lipid when priming was combined with sprouting (Table 5). A plausible explanation could be that the loss of carbohydrates through seed metabolism and respiration caused an increase in other nutrients, such as crude fats. Nonetheless, an increase in fat after sprouting has also been reported by Coello et al. [51]. Changes in fat content during sprouting depend on the crop type, genotype, and germination conditions such as temperature, soil, and water pollution [59], which can also affect its bioaccumulation in the seed.

#### 3.3.3. Crude Fiber and Carbohydrate Content

The crude fiber content of *MO* seeds was 7.63 ± 0.63*%*. Seed priming only treatments, that is, SW and SN, reduced the crude fiber content as compared with the control. A further reduction of 43.7% in crude fiber content was observed when seed priming was combined with sprouting (SWS and SNS). Carbohydrate content was 14.97 ± 0.56*%* and 15.26 ± 0.47*%* for SN and SW, respectively, which is a 38.6%–41.1% increase when compared with the control (NSNS). Conversely, combining seed priming with sprouting treatments reduced the carbohydrate content to 7.19 ± 2.39*%* (SWS) and 8.52 ± 0.96*%* (SNS), which is approximately a 21%–33% reduction when compared with NSNS. Carbohydrates in the form of starch in the seed endosperm are hydrolyzed in the aleurone layer by hydrolytic enzymes such as *α*‐amylase for energy production to support germination [60]. Hence, possibly contributing to the reduction in carbohydrate content of sprouted *MO* seed flour. This study’s results are in line with the literature, such as Matthew et al. [61], who also reported a reduction in carbohydrate content after sprouting *MO* seeds.

#### 3.3.4. Monosaccharide Content

As seen in Table 5, HPLC analysis identified four (4) monosaccharides, which are glucose, xylose, mannose, and arabinose, for *MO* seed flour. Glucose was identified as the dominant monosaccharide for flour made from SWS 1.65 ± 0.04 and 1.97 ± 0.09 g/100g for SNS. The increase in glucose content after exposure to an alkali medium is an indication of a tolerance to alkali stressors [62], and this was observed for SN treatment. Parallelly, a 20%–25.74% increase in total soluble sugars was observed when rice seeds were subjected to abiotic stressors [28]. SWS was the only treatment that significantly impacted the xylose content by reducing it from 0.80 ± 0.06 to 0.67 ± 0.00 g/100g while increasing the mannose content from 0.97 ± 0.02 to 1.14 ± 0.00 g/100g. The xylose and mannose content of all other treatments was not different from the control (NSNS). Overall, there was a progressive decrease in arabinose content across all the *MO* seed treatments when compared with the control. Hydropriming (SW) had a 5% reduction effect on arabinose content, while priming with 0.5% NaOH (SN) resulted in a 13% reduction. When priming treatments were combined with sprouting, there was a 28% or 30% reduction in arabinose content for SNS and SWS seed flour, respectively. A similar reduction in arabinose was reported by Saa et al. [14] when *MO* seeds were soaked, sprouted, and roasted. The differential reduction in arabinose content depicts the effect of seed priming and sprouting on the cell wall dissolution of *MO* seeds. Data from this current study also suggest that seed priming and/or sprouting of *MO* seeds supports the accumulation of glucose and mannose, while arabinose and xylose are leached out or utilized during seed germination. Nevertheless, caution must be exercised by consumers with Type II diabetes since the increase in flour glucose content can spike blood glucose levels, which is detrimental to their health.

#### 3.3.5. Energy Content

All *MO* seed treatments increased the energy value of the resultant seed flour. Specifically, *MO* flour made from SWS had the highest value for energy (530.60 ± 4.21 kcal/100g), which represents a 4.1% increase when compared with the control, while SW, SN, and SNS treatments contributed to a 1%, 2%, and 2.5% increase in energy values, respectively.

#### 3.3.6. Mineral Content

Minerals are essential nutrients that contribute significantly to health and well‐being. The dominant mineral content identified in untreated *MO* seed flour was phosphorus (1184 ± 0.01 mg/100g), then calcium (336.67 ± 0.00 mg/100g), followed by iron (12.53 ± 0.39 mg/100g) and finally zinc (1.44 ± 0.19 mg/100g) (Table 5). These findings are similar to studies reported by Taiwo Olagbemide and Alikwe [63] on *MO* seeds. In this study, the phosphorus content of *MO* seeds is higher than that reported for popular cereals such as brown top millet, that is, 280.8 mg/100g [64], soya, that is, 696.51–763.81 mg/100g [65], and sesame seeds, that is, 342–641 mg/100g [66]. This suggests that *MO* seed flour can potentially be a good source of phosphorus, which can improve the bone health of consumers. In terms of *MO* seed treatments, the differential results in mineral content were based on the media used for seed priming. Based on the mean values (Table 3), phosphorus, calcium, iron, and zinc concentrations were not significantly (*p* < 0.05) different between SW and the control; however, there was an 81% reduction in iron, an 80% reduction in phosphorus, a 59% reduction in zinc, and a 13% reduction in calcium when SN treatment was applied. The alkaline medium may have contributed to the increased rate of leaching of these minerals, hence their drastic loss. Similarly, when seed priming and sprouting treatments were combined, there was a reduction (*p* < 0.05) among all mineral content, especially when compared with the control (Table 5). However, between the sprouting treatments, there were no significant variations in the mineral content except for iron, which was reduced by 53% for SNS when compared to SWS. The reduction in the iron content of the sprouted seed flour reflects its utilization in the sprouting process. Iron is remobilized by the seed for radicle formation and emergence, phosphorus forms part of the adenosine triphosphate (ATP) cycle to provide energy for germination, calcium plays a role in cell wall development and rigidity, and zinc is involved in the translocation of iron for shoot development. Some studies have also documented a reduction in mineral content following sprouting for various sprouting durations [67–69]. To improve upon the mineral quality of sprouts, Park et al. [67] suggest priming seeds in mineral‐dense media before sprouting, and the author highlights varying levels of success in improving the iron content of alfalfa sprouts. This can be an alternative approach that can be explored to overcome the reduction in mineral quality following the sprouting of *MO* seeds.

#### 3.3.7. Antinutrient Content

The changes in the antinutritional content of *MO* seeds during seed priming and germination are represented in Table 5.

##### 3.3.7.1. Phytic Acid

Phytic acid is one of the major antinutrients of concern, especially in plant‐sourced foods, due to its divalent cation interaction with minerals such as iron, zinc, magnesium, and calcium, hence forming insoluble complexes which significantly reduce their bioavailability and assimilation during food digestion [70, 71]. *MO* seeds had a high phytic acid content, that is, 70.00 ± 0.00 mg/100g for untreated seeds when compared to all the treated *MO* seed flour in this study (Table 5). Other studies on brown top millet [64], rice [72], and commonly consumed Canadian pulses [73] also report high phytic acid content, that is, 16.4 mg/g for brown top millet, 20.1 g/kg for rice, and 8.55–22.91 mg/g for Canadian pulses. Nonetheless, the phytic acid content for untreated *MO* seeds does not exceed the maximum concentration range of 250–500 mg/100 g for safe human consumption [74]. This current study reveals a 19% and 28% reduction in phytic acid of *MO* seeds when SW and SN treatments were utilized, respectively. A similar reduction trend was reported by Matthew et al. [61] and Saa et al. [14] after soaking *MO* seeds for 24 h. The authors ascribed the reduction in phytic acid to the leaching of phytate into the soaking medium. Furthermore, among all the treatments investigated in this study, hydropriming and sprouting resulted in the highest attenuation (42.8%) in phytic acid. During germination, dephytinization of phytic acid is carried out by phytase, which in turn makes phosphorus available back for absorption and utilization in the ATP cycle for energy release to support germination. A reduction in phytic acid is necessary to improve the bioavailability of iron, zinc, and other dietary components; thus, SWS treatment of *MO* seeds causes a reduction in phytic acid and can improve its nutritional contribution when utilized in plant‐based meals.

##### 3.3.7.2. Alkaloids

Alkaloids are another group of antinutrients that reduce the bioavailability of vitamins. Neurodegenerative and digestive illnesses have been associated with the regular consumption of food containing alkaloids that exceed 20 mg/100 g sample [75]. The alkaloid content for untreated *MO* seeds was 4.08 ± 0.03 mg/100 g, which is below the toxicological limits for safe consumption. A reduction in alkaloid content was only observed for SW‐ and SWS‐treated *MO* seed flour. This reduction represents a 28%–33% loss in alkaloid concentration when compared with the untreated *MO* seeds. In contrast, and based on the Tukey comparison of means, SN and SNS did not significantly reduce the alkaloid content since the alkaloid content was comparable to the control.

##### 3.3.7.3. Oxalate

Oxalate content in untreated *MO* seed flour was 2.7 ± 0.01 mg/100 g, which is lower than that reported by Shi et al. [73] for many popularly consumed legumes such as peas, lentils, fava beans, chickpeas, soybeans, and common beans. Based on the results from this study, it is possible to significantly reduce the oxalate content of *MO* seed by 30%–76% using the seed priming and/or sprouting approaches outlined. Specifically, SW and SWS treatments can reduce the oxalate content by 30%–39.9%, respectively, while SNS and SN can reduce the oxalate content by 60%–76%, respectively. The reported reduction in oxalate content can be attributed to the leaching of soluble oxalate into soaking media; hence, soaking media have a critical role in reducing oxalate content in *MO* seeds. This is of great significance because ingestion of a minimal dose of 5 g oxalic acid in crystal form or solution can be associated with renal damage due to the formation of insoluble calcium oxalate [76, 77]. The consumption of plant‐based food is increasingly popular due to a surge in vegan diets; hence, simple yet efficient methods such as those presented in this study can effectively reduce the oxalate in *MO* seeds and possibly contribute to increased consumption.

##### 3.3.7.4. Tannin

Tannins are ubiquitous secondary metabolites of plant origin which can form complexes with proteins and digestive enzymes. They can also be described as antinutrients since they interfere with protein bioavailability and digestibility as well as the assimilation of vitamin B_12_ and Fe^+2^ [78]. Nonetheless, recent epidemiological and pharmacological studies have highlighted the anti‐inflammatory, antioxidative, and antimicrobial potential of tannins based on their in vivo or in vitro biological effects [79] within safety limits. In this study, tannins were detected in untreated and treated *MO* seed flour. The tannin content in untreated *MO* seed flour was 2.64 ± 0.10 mg/100 g, and SW treatment was the only treatment that caused a 9.8% reduction in tannin content. Similar reduction trends in tannin content were reported by Bhuvaneshwari et al. [80] for hydroprimed millet and Adeleke et al. [35] for Bambara groundnut. This reduction was attributed to the leaching of water‐soluble tannins; hence, water can cause a reduction in the tannin content of seeds [78]. Conversely, a 25% increase was detected when *MO* seeds were treated by SN. This can be attributed to the seed’s response to oxidative stress triggered by the slight alkalinity of the soaking media. Correspondingly, a 7.8%–18.9% increase in tannin was detected in the seed flour when *MO* seeds were treated by SWS and SNS, respectively. In a review of similar studies, an increase in tannin content after sprouting was reported for Amaranthus seeds [79], rice [72], sorghum, and fonio seeds [81]. Abiotic stressors at the seed imbibition stage and the later phases of germination can trigger the gene expression and activity of defense enzymes such as PAL, which are upregulated to play a vital role in reducing seed oxidative stress and cell damage caused by the release of free radicals [9]. These enzymes catalyze the biosynthesis of phenylpropanoid compounds to release phenolic compounds such as tannins [51]. The culmination of all these activities could have contributed to the resynthesis and accumulation of tannins in sprouted *MO* seed flour.

### 3.4. Effect of Seed Priming and Sprouting on the Functional Characteristics of *MO* Seed Flour

The functional characteristics of flour were investigated to understand the impact of *MO* seed treatment on the functional, phenolic, and antioxidative characteristics of the resultant flour and to ascertain the possible food application. Table 6 depicts the findings on the functional characteristics of *MO* seed flour.

**Table 6:** Functional properties of *MO* seed flour

**Parameters**	**Treatments**					
	**NSNS**	**SW**	**SN**	**SWS**	**SNS**	**P value**
Water holding capacity (g/mL)	1.42±0.06^c^	1.47±010^bc^	1.44±0.05^bc^	1.92±0.10^ab^	2.06±0.58^a^	<0.05
Oil holding capacity (g/mL)	2.15±0.22^a^	2.41±0.33^a^	1.97±0.15^a^	1.97±0.39^a^	2.34±0.20^a^	>0.05
Bulk density (g/cm^3)^	0.47±0.01^b^	0.54±0.01^a^	0.55±0.00^a^	0.54±0.01^a^	0.53±0.00^a^	<0.05
pH	5.56±0.05^c^	5.86±0.02^b^	6.15±0.03^a^	5.84±0.12^b^	5.69±0.05^bc^	<0.05

*Note:* Data represent the mean of three independent experiments (*n* = 3). Means with different superscripts in the row indicate significant differences (*p* < 0.05)

Abbreviations: NSNS (Control): Not soaked, not sprouted; SW: soaked in water; SN: soaked in 0.5% NaOH solution; SWS: soaked in water with sprouting; SNS: soaked in 0.5% NaOH solution with sprouting

#### 3.4.1. WHC and OHC

Flour made after SW treatment had a WHC of 1.47 ± 0.10 g/mL, significantly similar to the WHC, that is, 1.44 ± 0.05 g/mL of SN treatment (Table 6). However, when seed priming was combined with sprouting treatments, there was an increase in WHC ranging from 1.92 ± 0.10 to 2.06 ± 0.58 g/mL for SWS and SNS, respectively. This represents a 35%–45% increase in the WHC when compared to NSNS. This implies that the WHC of *MO* seed flour can be influenced by seed priming and sprouting treatments. An increase in WHC could be attributed to an increase in protein content and a change in the structure of protein upon germination. A change in the native structure of proteins can be induced by proteolysis initiated by germination, leading to the exposure of hydrophilic sites of the protein to enhance the flour’s WHC [82, 83]. Additionally, during germination, polysaccharide molecules break down; hence, the sites for interaction with water and holding water are increased. Other studies also reported a 7.84% elevation in WHC of sorghum [84], a 5.88% increase for pigeon pea [85], and 15%–73.3% elevation for lentil and horse gram, respectively [86] after germination.

The OHC of treated *MO* seed flour ranged between 1.97 ± 0.39 and 2.41 ± 0.33 g/mL, and the means were not significantly (*p* > 0.05) different from the control. According to this, the oil retention capacity of *MO* seed flour remained consistent across all seed treatments, indicating that the hydrophobic properties of *MO* seed proteins did not significantly vary.

#### 3.4.2. Bulk Density of Treated and Untreated *MO* Seed Flour

Flour bulk density is a critical factor during decisions on flour handling, processing, and end‐use. The bulk density of flour from untreated *MO* seed was 0.47 g/cm. In this study, seed priming and sprouting significantly increased (*p* < 0.05) the bulk density of the resultant flour. This represents a 12%–17% increase in the bulk density of treated *MO* seed flour when compared with untreated *MO* seed flour (Table 6). A similar observation of increased bulk density was reported for sprouted wheat [87]. Generally, the increase in bulk density can be attributed to the moisture content and the WHC of the seeds. The seeds’ treatment has a direct impact on these variables. Corroborating evidence in this study has shown a 35%–45% increase in the WHC of the primed and sprouted *MO* seeds, which consequently increased the bulk density of their resultant flours. This implies that a large quantity of flour can occupy a small volume of a package, which has a positive implication for efficient packaging and transportation. Regarding formulations, the high bulk density of treated *MO* seed flour can have possible applications as a thickener in food.

#### 3.4.3. pH of Treated and Untreated *MO* Seed Flour

The pH of *MO* seed flour was dependent on the seeds’ treatment before flour processing. Specifically, within the priming only treatment, SW treatment had a pH of 5.86, which was significantly different (*p* < 0.05) from SN treatment, which had a pH of 6.15. Compared to the control (pH 5.56), this increase in pH can be primarily ascribed to the pH of the priming media. Conversely, combining hydropriming or alkaline‐priming with sprouting resulted in similar mean values for pH, that is, 5.84 and 5.69 for SWS and SNS, respectively. Generally, sprouting resulted in a decrease in the pH. This was not significant for SWS; however, the decrease was significant (*p* < 0.05) for SNS (i.e., from 6.15 for SN to 5.69 for SNS). Spontaneous fermentation coupled with an increase in the activity of hydrolyzing enzymes during seed priming and sprouting can facilitate the release of organic acids, following the breakdown of complex compounds; hence, a slight decrease in the pH of the resultant flour was observed. In this context, similar results were highlighted by Dhillon et al. [87], who also reported a decrease in pH of flour made from sprouted wheat grains.

#### 3.4.4. Total Phenolics Content and DPPH Free Radical Scavenging Activity

*MO* seed extracts have been extensively researched as an ayurvedic plant, which directly or indirectly contributes to alleviating diseases caused by mutagenic and carcinogenic cells generated from oxidative assault on DNA [88, 89]. The health‐promoting potential of *MO* seed extracts can be attributed to the individual or combined effects of its constituents, that is, minerals and phytochemicals (flavonoids and phenolic compounds), which are inhibitors of carcinogenesis via redox reactions [90]. Therefore, to determine the potential bioactivity of the flour from *MO* seeds, the total phenolic content (TPC) was determined, and the in vitro antioxidative potential of the methanolic extracts was quantified based on DPPH free radical scavenging activity (Table 7).

**Table 7:** Bioactive properties of treated *MO* seed flour.

**Parameters**	**Treatments**				
	**NSNS**	**SW**	**SN**	**SWS**	**SNS**
Total phenolics (mgGAE/100g)	8.94 ± 0.20^c^	11.87 ± 0.07^a^	8.81 ± 0.16^c^	9.96 ± 0.19^b^	10.18 ± 0.30^a^
DPPH %	6.02 ± 1.13^c^	9.54 ± 0.87^b^	6.14 ± 1.22^c^	10.38 ± 1.33^b^	13.65 ± 0.65^a^

*Note:* Data represent the mean of three independent experiments (*n* = 3). Means with different superscripts in the row indicate significant differences (*p* < 0.05).

Abbreviations: NSNS (control), not soaked, not sprouted; SW, soaked in water; SN, soaked in 0.5% NaOH solution; SWS, soaked in water with sprouting; SNS, soaked in 0.5% NaOH solution with sprouting.

The highest phenolic content (11.87 ± 0.07 mg GAE/100g) was recorded by flour made from the SW treatment. This was followed by flour made after SNS treatment (10.18 ± 0.30 mg GAE/100g), while the control recorded the lowest (8.94 ± 0.20 mg GAE/100g). TPC increased by 11.4%–13.8% after SWS and SNS treatments. This can be attributed to the expression of PAL which is an enzyme that regulates the biosynthesis of phenolic compounds through the phenylpropanoid pathway. Additionally, polyphenol synthesis and accumulation are generally stimulated in response to biotic or abiotic stresses such as germination. Studies such as Coello et al. [51] also reported an increase in phenolic content of *MO* seeds following sprouting and generally attribute this phenomenon to the germination conditions.

Generally, an increase in TPC can reflect an increase in the antioxidative potential of the seed [91]. The DPPH free radical scavenging activity was expressed as a percentage (%). The highest DPPH scavenging activity was 13.65 ± 0.65*%* and was registered by flour produced by SNS treatment. The lowest (6.0 ± 1.13*%*) was observed for the control. This suggests that combining seed priming and sprouting can increase the concentration of extractable antioxidant from *MO* seeds, which can be further used in pharmacological studies. Proof of this concept can be further investigated through in vivo studies to elucidate the mechanism and biochemical processes of the antioxidative potential of sprouted *MO* seed flour.

### 3.5. Microbiological Quality of Treated *MO* Seed Flour

#### 3.5.1. Microbial Assessment of Treated and Untreated *MO* Seed Flour

The microbial load of sprouted seeds is of major food safety concern, mainly due to the conditions under which the sprouts are produced. Sprouts are produced under warm, humid conditions, which are also ideal for the growth of pathogens [17]. Despite its health benefits, sprouts have been implicated in ongoing food safety issues [92, 93] such as outbreaks associated with *Salmonella*, pathogenic *E. coli* (O157:H7), and *S. aureus* [94]. Hence, it is important to determine the level of microbial safety of the sprouts before consumption; this data is displayed in Table 8. This study followed good manufacturing practices (GMPs) for sprout production outlined by the European Sprouted Seeds Association (ESSA) [95], and *MO* seeds were pretreated with 200 ppm of NaOCl for 5 min before sprout production as a mitigation measure to reduce microbial load. Additionally, the optimal seed priming conditions were determined to avoid excessive priming, which leads to the accumulation of microbes and fermentation of seeds [96]. Furthermore, the seeds were monitored regularly, and seeds that showed signs of mold growth during storage or germination were discarded to prevent contamination of the batch. Therefore, mold growth was controlled throughout the production process and increments in mold counts were marginal (Table 8). As shown in Table 8, yeast, *E. coli*, and *S. aureus* were not detected for all flour samples when NaOCl was used as a disinfectant, which is similar to reports by Gilbert et al. [97] for the use of NaOCl for disinfection of various seeds for sprout production. This means that the sanitation protocols and disinfection methods in this study were effective in controlling the growth of these pathogens. Nonetheless, the APC of all the flour ranged between 2 and 4 log CFU/g. An increase of approximately 1–2 log CFU/g was observed during the seed priming and sprouting phases of production. The sprouting phase of production had the highest APC value when compared with the seed priming phase. Sprouting is generally conducted under high humidity (> 90%) and warm temperatures (20°C–25°C), which is conducive for aerobic bacteria to thrive [93]. Additionally, the nutrient‐rich extrudates from the sprouts can provide nutrients to support bacterial growth; hence, it was expected that the coliform counts would increase under sprouting conditions. Nevertheless, the APC for sprouted flour (SWS and SNS) was within acceptable levels (5 log CFU/g) as prescribed by the ESSA [95]. Concerning seed sprouts and processing with NaOCl, it was observed that prolonged exposure of *MO* seeds to the sanitizing solution was harmful to the seeds (acidity led to off‐odors, eventual decay, and no germination); hence, the duration of presanitation measures is critical to ensure the viability of *MO* seeds for sprout production.

**Table 8:** Microbial evaluation of *MO* seed flour after pretreatment of seeds with 200 ppm of sodium hypochlorite (NaOCl).

**Production phase**	**Sample code**	**Log CFU/g**				
		**APC**	**Yeast**	**Mold**	***E. coli* (O157:H7)**	***S. aureus***
Control	*NSNS*	2.98	nd	nd	nd	nd
Seed priming phase	*SW*	3.07	nd	1.18	nd	nd
	*SN*	2.67	nd	1.77	nd	nd
Sprouting phase	*SWS*	4.87	nd	1.04	nd	nd
	*SNS*	4.86	nd	1.04	nd	nd

*Note:* All seeds were disinfected with 200 ppm NaOCl for 5 min before processing.

Abbreviations: APC, aerobic plate count; nd: not detected; NSNS (control), not soaked, not sprouted; SN, soaked in 0.5% NaOH solution; SNS, soaked in 0.5% NaOH solution with sprouting; SW, soaked in water; SWS, soaked in water with sprouting.

### 3.6. Effect of Seed Priming and Sprouting on Sensory Characteristics of Treated *MO* Seed Flour

**Figure 4:**
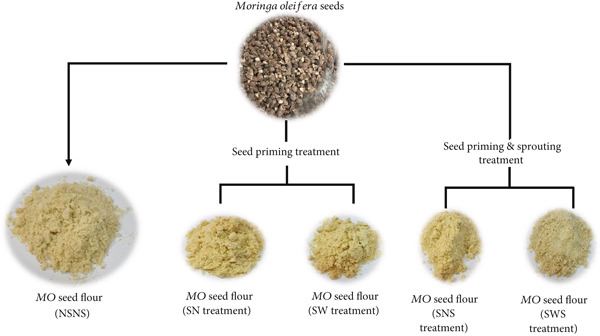
*MO* seed flour from seed priming and/or sprouting treatment NSNS: Control; SWS: Soaked in water and sprouted; SNS: Soaked in 0.5% NaOH and sprouted; SN: Soaked in 0.5% NaOH only; SW: Soaked in water only.

A QDA was conducted to identify the qualitative and quantitative sensory attributes of treated and untreated *MO* seed flour in its raw state (Figure 4). Four (4) main attributes and twenty‐one (21) sensory descriptors and lexicons (Table 2) were identified, described (Table 3), and quantitatively evaluated. The mean sensory ratings and ANOVA are presented in Table 9.

**Table 9:** The effect of *MO* seed treatment on sensory attributes.

**Sensory attribute**	***MO* seed treatment**				
	**NSNS**	**SW**	**SN**	**SWS**	**SNS**
*Aroma*					
Herby	5.00^a^	5.06^a^	5.09^a^	4.05^b^	4.36^c^
Nutty	4.72^c^	4.66^c^	4.81^bc^	5.06^a^	5.04^ab^
Rancid	2.81^e^	3.09^c^	3.03^d^	3.53^a^	3.39^b^
Fermented	2.15^b^	2.39^a^	2.33^a^	2.36^a^	2.28^a^
*Flavor*					
Rancid	2.67^c^	3.18^a^	2.78^bc^	3.30^a^	2.84^b^
Astringent	3.87^ab^	4.03^a^	3.93^ab^	2.50^c^	3.28^b^
Nutty	3.84^b^	3.66^b^	3.73^b^	4.64^a^	4.15^ab^
Beany	3.87^c^	4.05^ab^	4.00^bc^	4.13^a^	3.98^bc^
Chili ^∗^	1.84^a^	1.98^a^	2.14^a^	1.93^a^	1.90^a^
Sour	2.36^a^	2.33^a^	2.30^a^	1.57^b^	2.03^ab^
Sweet	4.09^c^	4.01^cd^	3.72^d^	5.64^a^	4.78^b^
Bitter	5.21^a^	5.57^a^	5.24^a^	3.10^b^	4.18^c^
*Texture*					
Oily	3.18^c^	3.28^bc^	3.33^abc^	3.77^a^	3.71^ab^
Smooth	5.87^b^	5.48^d^	6.00^a^	5.12^e^	5.72^c^
Gritty	3.78^c^	4.18^b^	3.78^c^	4.69^a^	3.96^c^
Melty	4.93^ab^	4.72^b^	5.21^a^	4.73^b^	4.72^b^
*Aftertaste*					
Astringent	6.27^a^	3.99^b^	3.84^c^	3.63^d^	3.81^c^
Oily	3.18^b^	3.20^b^	3.29^b^	3.51^a^	3.42^a^
Sweet	4.52^c^	4.97^b^	4.63^c^	5.33^a^	5.27^a^
Bitter	4.97^a^	5.03^a^	4.92^a^	4.12^b^	4.48^c^
Chili	1.84^ab^	2.00^a^	1.87^a^	1.31^b^	1.85^a^

*Note:* Data represent the mean of three independent experiments (*n* = 3). Means with different superscripts in the row indicate significant differences (*p* < 0.05). Chili ^∗^ has a *p* value >0.05.

Abbreviations: NSNS (control), not soaked, not sprouted; SN, soaked in 0.5% NaOH solution; SNS, soaked in 0.5% NaOH solution with sprouting; SW, soaked in water; SWS, soaked in water with sprouting.

The results show that amongst the twenty‐one (21) sensory descriptors identified and evaluated, only one (1) descriptor was not statistically significant with respect to its perceived intensity, that is, chili flavor. However, significant differences were observed in the intensities of all the evaluated descriptors due to the various treatments given to the *MO* seeds. Based on Tukey difference of mean analysis (Table 9), SWS and SNS had similar intensities for nutty and fermented aromas and sour flavor, oily texture, and melty mouthfeel, and oily and sweet aftertaste. Primed seeds (i.e., SW and SN) had similar intensity profiles for herby, nutty, and fermented aroma and flavor, oily, bitter, and chili aftertaste.

The bioprocess of germination initiates seed metabolism, resulting in the catabolism and degradation of macronutrients and some antinutritional compounds, which can impart the sensory characteristics of the resultant flours. The culmination of all the sensory attributes was indicated in the overall flavor intensity. To observe the effect of seed treatment on the sensory attributes of *MO* seed flour, the overall flavor with respect to bitterness, sweetness, sour, beany, astringent, rancid, chili, and nutty flavors is displayed in the spider diagram in Figure 5.

Figure 5The effect of *MO* seed treatment on overall flavor intensity. NSNS (control), not soaked, not sprouted; SW, soaked in water; SN, soaked in 0.5% NaOH solution; SWS, soaked in water with sprouting; SNS, soaked in 0.5% NaOH solution with sprouting. (a) Control vs. SW. (b) Control vs. SN. (c) Control vs. SWS. (d) Control vs. SNS.

**Figure:**
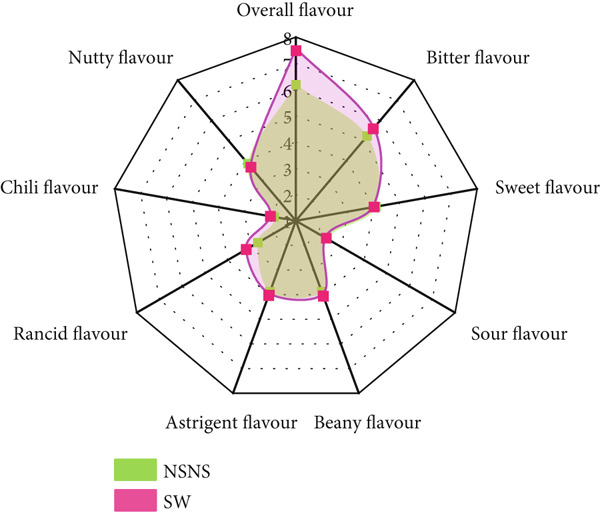
(a)

**Figure:**
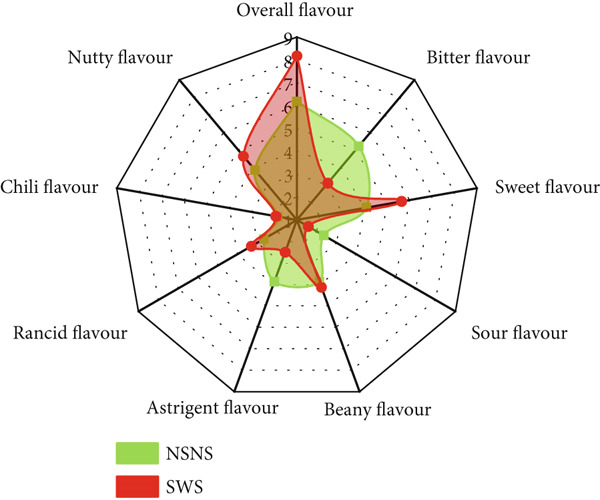
(b)

**Figure:**
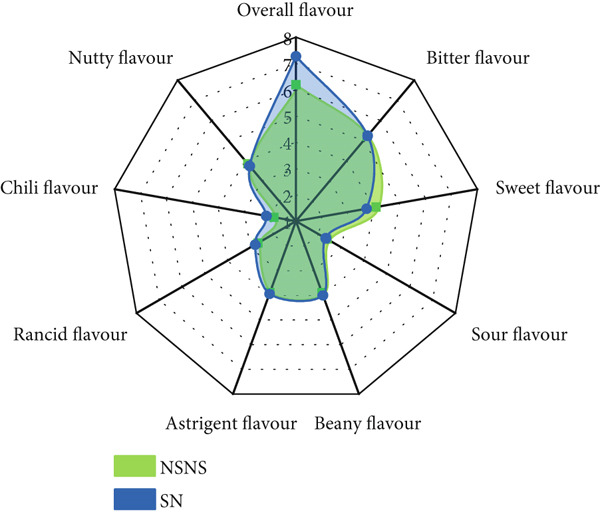
(c)

**Figure:**
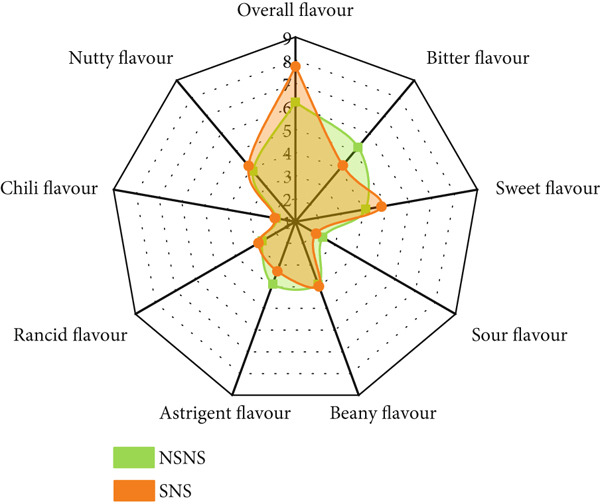
(d)

#### 3.6.1. PCA of Sensory Product Map of *MO* Seed Flour

A PCA was conducted to clarify the contribution of seed treatments to the sensory profile of *MO* seed flour (Figure 6). Four principal components (PC1–PC4) were extracted, and eigenvalues accounted for by each principal component were 15.847, 3.682, 2.615, and 0.856, respectively. The first component (PC1) explained 68.9% of the variance, while the second (PC2) explained 16.01% of the variance in the sensory profile of treated *MO* seed flour. The cumulative variance contribution of PC1 and PC2 was 84.91%, and it was interpreted as 84.91% of the variation in the sensory profile of the *MO* seed flour was due to the seed priming and sprouting treatments. The dots on the score plot (Figure 6a) represent the five different seed treatments, while the direction and length of the lines on the loading plot (Figure 6b) represent the orientation of the sensory attributes as influenced by the seed treatment. It can be seen from Figure 6a that NSNS was separated from all the other seed treatments in the fourth quadrant, indicating an obvious difference in sensory attributes of the seed flour. However, SW and SN were clustered in the first quadrant, indicating similar sensory attributes. Alternatively, SWS and SNS were separated into the second and third quadrants, respectively. Regarding the sensory attributes, beany flavor, rancid flavor, sweet aftertaste, and aroma attributes such as fermented and rancid aroma had great contributions towards the sensory characteristics of SWS. Nutty and sweet flavors contributed predominantly to the sensory characteristics of *MO* seed flour from SNS treatment. While herby aromas, bitter, and sour flavor were identified with flour made from SW and SN. The control (NSNS) had an astringent aftertaste and a melty and smooth texture (Figure 6b). Generally, the sensory characteristics of sprouted seed flour are predominantly influenced by the biochemical changes occurring during sprouting. Specifically, the characteristic sweetness associated with SWS and SNS can be attributed to starch degradation by *α*‐amylase. These enzymes increase in quantity and activity during germination to break down complex sugars into simple sugars such as glucose, which enhances the sweetness of the seed flour. Corroborating data from the proximal content of sprouted *MO* seed flour (Table 5) shows a 5%–25.47% increase in glucose content compared with the control, which inevitably influences the sweet perception of the sprouted *MO* seed flour. Secondly, the nutty aroma and flavors associated with sprouted *MO* seeds can be due to the presence and activity of lipases, which hydrolyze lipids such as linoleic acid to release free fatty acids and volatile compounds such as hexanal [98]. This gives sprouted seeds their characteristic nutty aroma. Furthermore, lipoxygenases (LOXs) catalyze the oxidation of fatty acids, resulting in the formation of volatile compounds such as short‐chained aldehydes and ketones, which can be perceived as rancid or beany [99]. These biochemical changes collectively can influence the sensory profile of sprouted *MO* seeds. Finally, proteases break down proteins into peptides, which subsequently undergo Maillard reactions due to the interaction between peptides, lipids, and simple sugars during the drying process. This produces nutty aromas and flavors that are characteristic of sprouts from oil seeds. Based on these concepts, it can be concluded that the 5%–9% increase in protein and fat content of sprouted *MO* seeds, the presence of hydrolyzing enzymes, coupled with the flour processing conditions, contributed to enhancing the sensory characteristics of sprouted *MO* seed flour.

Figure 6Principal component analysis of (a) score plot and (b) loading plot of sensory data from treated *MO* seed flour; *F*1 : *F*2 = 84.91*%*. NSNS (control), not soaked, not sprouted; SW, soaked in water; SN, soaked in 0.5% NaOH solution; SWS, soaked in water with sprouting; SNS, soaked in 0.5% NaOH solution with sprouting; A.T, aftertaste.

**Figure:**
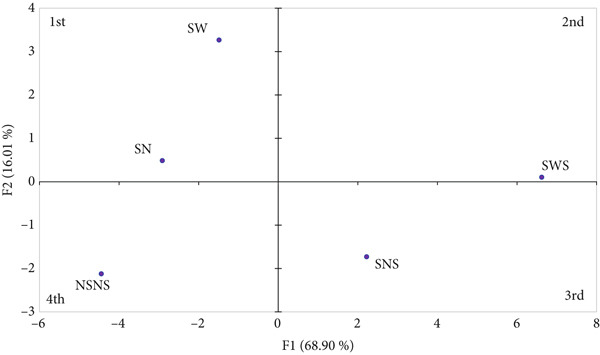
(a)

**Figure:**
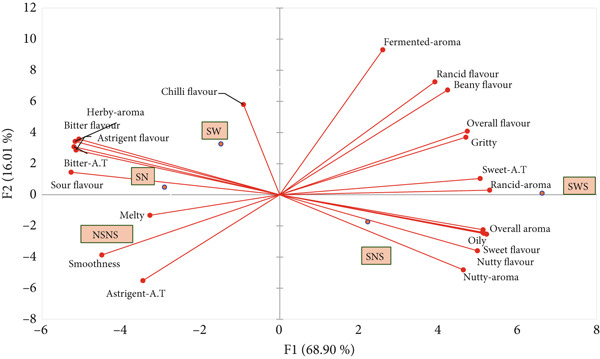
(b)

Conversely, the bitter, astringent, and sour flavors and the herby aroma of *MO* seed flour after SW and SN treatments were atypical of the sensory characteristics of the *MO* seeds, which were similar to reports by Ani et al. [100]. This suggests that seed priming as a sole processing step is insufficient to influence the sensory attributes of *MO* seeds. Therefore, elucidating these biochemical changes is critical for optimal sprout quality and nutritional value. Hence, based on the study conditions outlined in this research, it is possible to enhance the sensory characteristics of *MO* seed flour, especially when seed priming is coupled with sprouting.

## 4. Conclusion and Future Direction

In this study, it has been shown that 9 h of hydropriming is sufficient to initiate the metabolic processes that support the germination of *MO* seeds. Hydropriming results in a 92% germination rate, which represents a 29.94% increase in germination rate when compared with alkaline‐primed seeds. Seed priming as a sole processing approach for *MO* seeds is insufficient to improve the quality and sensory appeal of *MO* seed flour. The major highlight in this study lies in combining seed priming with sprouting, which is an effective dual‐treatment strategy to reduce the phytic acid, alkaloid, and oxalate content of *MO* seed flour. *MO* seed flour with a protein content of 45% can be produced when seed priming is combined with sprouting. The astringent and bitter taste of *MO* seed flour is a major drawback for *MO* seed utilization in food applications. However, based on the protocols outlined in this study, seed priming with sprouting can impart sweet and nutty flavors to *MO* seed flour, which is a desirable trait for increased consumption. Additionally, the increase in the total phenolic and antioxidative activities of sprouted *MO* seed flour contributes to its bioactivity with potential health‐promoting benefits. *MO* seeds, although underutilized, have food potential with specific contributions towards SDG 2 and 3 (zero hunger, health, and well‐being). This study has thus emphasized the connection between seed physiology and the optimization of food ingredients, presenting a promising path for developing flour ingredients from *MO* seeds that aligns with sustainable nutrition goals. To the best of our knowledge, this bridge between seed biochemical modulation and food ingredient optimization has not been fully explored in literature, especially for *MO* seeds, so this study contributes to the existing knowledge in this field.

Nonetheless, future studies to expand this concept can delve into mineral seed priming and sprouting treatment, with a focus on retaining the mineral content of *MO* seed flour to improve its nutritional quality and future food application. Alternatively, enrichment or mineral fortification can be applied with critical evaluation of its impact on flour functional and organoleptic quality. Shelf life and stability studies delving into the optimization of packaging and storage conditions will contribute to the commercial viability and holistic valorization of *MO* seed through flour production.

## Conflicts of Interest

The authors declare no conflicts of interest.

## Funding

This research was financially supported by the University of Ghana Nestlé Scholarship for Research Excellence through Nestlé SA.

## Data Availability

The dataset supporting the conclusions of this article is included in this article.
